# Erythrodysesthesia: An Unusual Complication With Trastuzumab Monotherapy

**DOI:** 10.7759/cureus.20060

**Published:** 2021-11-30

**Authors:** Andee L Fontenot, Weldon J Furr, Ammar Husan, Muhammad W Khan

**Affiliations:** 1 Medicine, Louisiana State University Health Shreveport, Shreveport, USA

**Keywords:** dermatologic complication, hand foot syndrome, herceptin, erythema, trastuzumab, oncology, dermatology, palmar plantar erythrodysesthesia

## Abstract

Palmar-plantar erythrodysesthesia (PPE), more commonly known as hand-foot syndrome, is a dermatologic complication following chemotherapy with selective agents. In this article, we present the case of a young lady with stage III breast cancer who developed palmar-plantar erythrodysesthesia following treatment with Herceptin (trastuzumab), an unusual complication of this particular drug. From our review of literature, this is the second known occurrence of PPE secondary to trastuzumab monotherapy.

## Introduction

Hand-foot syndrome (HFS) is a common dermatologic complication following selective cytotoxic chemotherapeutic agents. It is characterized by erythema, pain, numbness, and often tingling of the cutaneous layer of the palms and soles [1]. The National Cancer Institute Common Terminology Criteria for Adverse Events version 5.0 (NCI-CTCAE v5.0) is frequently used to classify the severity of the hand-foot syndrome. Criteria for a grade 1 syndrome include minimal skin changes such as erythema, edema, or hyperkeratosis in the absence of pain. If skin changes increase to the extent of peeling, blisters, bleeding, or fissures along with pain and instrumental activities of daily living (ADL) being limited, criteria for a grade 2 syndrome have been reached. The distinction between grade 2 and grade 3 criteria is determined by skin changes being severe enough to interfere with self-care ADL [2]. Though several chemotherapeutic agents may be involved alone or in combination, trastuzumab (Herceptin), a monoclonal antibody against the human epidermal growth factor receptor 2 (HER2) tyrosine kinase receptor, is an exceedingly rare cause for such cutaneous toxicity when being used as monotherapy [3].

## Case presentation

Appropriate protocols were followed, and consent was obtained by all participants in this study.

A young, 28-year-old Caucasian female was referred to the oncologist after noticing a lump in her right breast. Mammography revealed two lesions in her right breast; a 1.9 × 2.0 × 1.4 cm solid lesion and a cystic lesion measuring 1.8 × 1.5 × 1.5 cm. Subsequent immunohistochemistry (IHC) diagnosed the patient with an estrogen and progesterone (ER/PR) negative, HER2/neu positive invasive ductal carcinoma. Genetic testing was negative for breast cancer susceptibility gene mutations one and two (BRCA) as well as TP53 gene mutation. A composite risk of mutation probability, using the Hughes model, assigned the patient a 7% risk. Magnetic resonance imaging (MRI) of her breasts revealed a 2.7 cm mass in the upper inner quadrant of her right breast. The American College of Radiology (ACR) Breast Imaging Reporting and Data System (BI-RADS) was six and two of her right and left breasts, respectively.

The patient proceeded to bilateral mastectomy and axillary node clearance. She had a grade 3 residual invasive ductal carcinoma of her right breast, with one out of 13 lymph nodes found positive for metastasis.

She was started on adjuvant chemotherapy and radiotherapy thereafter. A total of 13 cycles (out of 17) were given, with the initial six cycles comprising of docetaxel, carboplatin, pertuzumab, and trastuzumab (first cycle at 2 mg/kg every week and subsequent cycles at 6 mg/kg every third week); each chemotherapy cycle was followed by subcutaneous Neulasta (6 mg). The latter seven cycles of chemotherapy comprised only of trastuzumab (at 6 mg/kg every three weeks). She appeared to be tolerating this regime well up to her 13th cycle.

**Figure 1:**
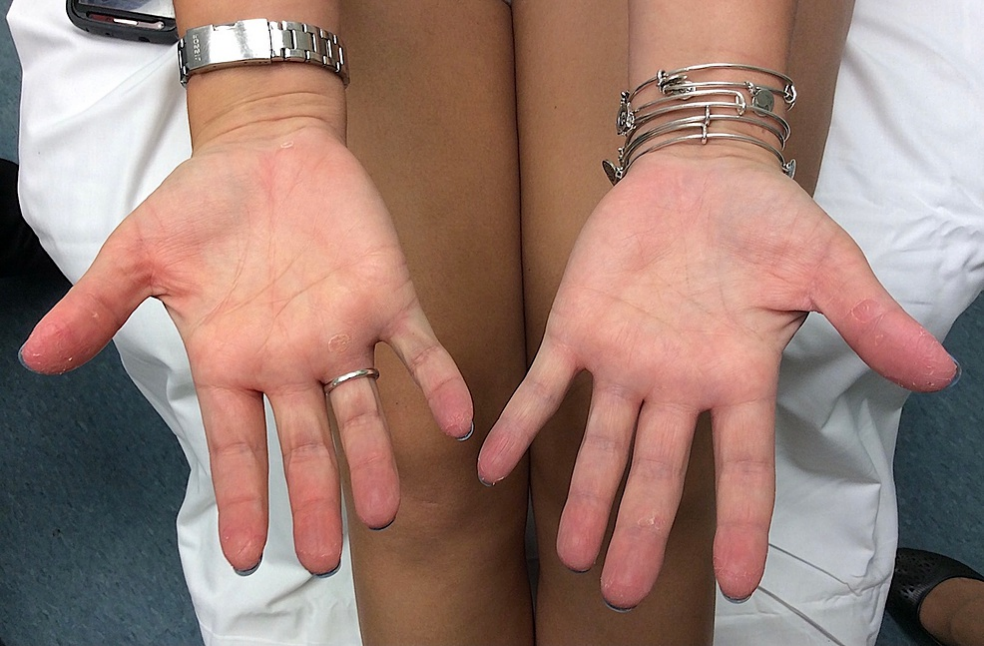
Cutaneous desquamation of the palms.

**Figure 2:**
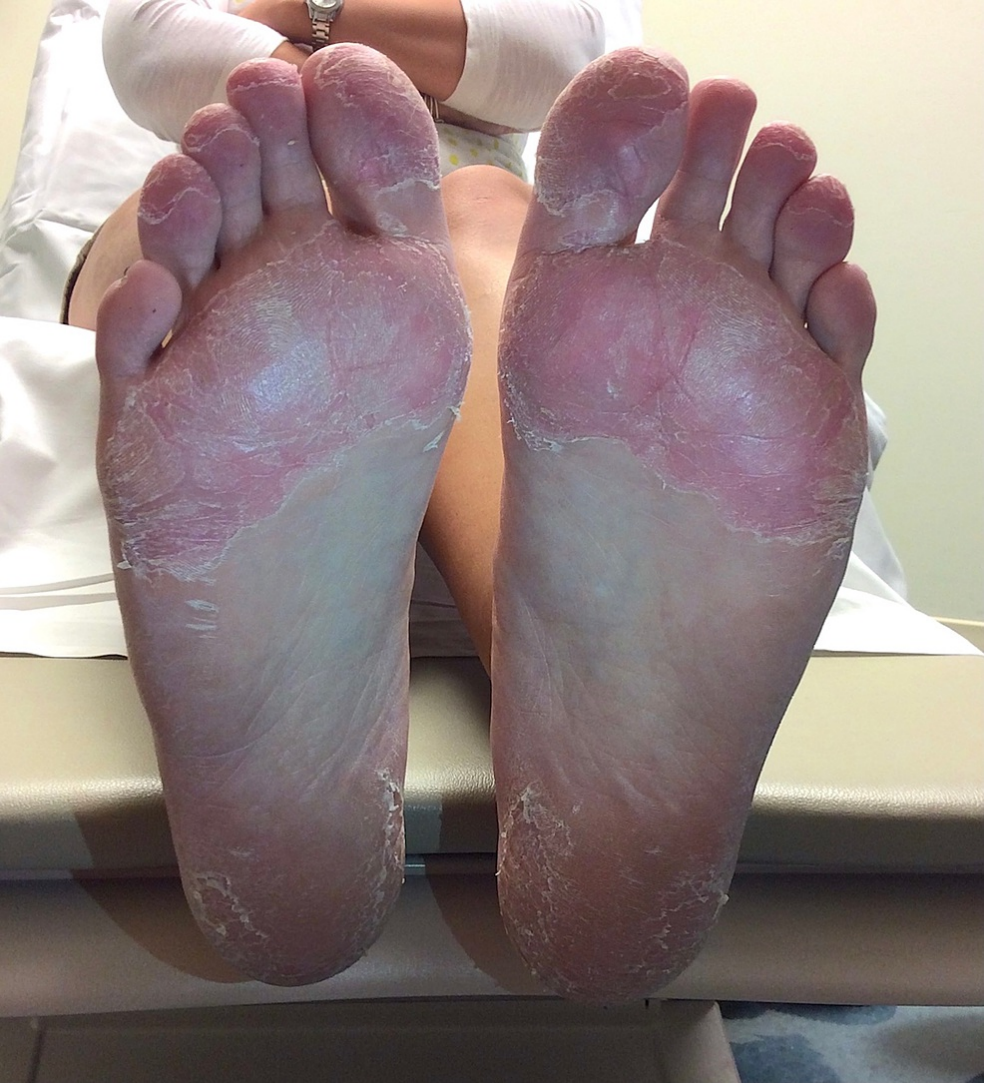
Cutaneous desquamation of the soles.

Trastuzumab administration proceeded uneventfully until a few days after her scheduled 14th cycle of chemotherapy, when she presented with superficial, dry, desquamation on the soles of her feet and palms of her hands with no interference of her daily life activities (Figures 1, 2). She had erythema and mild numbness in the tips of her fingers bilaterally. She had no associated complaints of tingling or tenderness and was hence diagnosed as a grade 1 palmar-plantar erythrodysesthesia in accordance with the National Cancer Institute grading (NCI).

She had not taken any new medication in the past few months aside from her routine trastuzumab. The patient was conservatively managed and counseled to the frequent use of emollients and moisturizers, however, a urea exfoliation product was not used. She was also advised to avoid extreme temperatures, friction, and tight-fitting shoes or gloves. Her chemotherapy was put on hold, and radiotherapy continued as scheduled. Her symptoms began to improve despite continued radiation therapy and resolved with a continued delay in trastuzumab therapy. Trastuzumab was then reintroduced after six weeks without any recurrence of erythrodysesthesia.

## Discussion

The National Cancer Institute (NCI) describes hand-foot syndrome (HFS), also called palmar-plantar erythrodysesthesia (PPE), as a condition marked by varying degrees of swelling, pain, tingling, numbness, vesiculation, or erythema predominantly of the hands or feet due to the cutaneous toxicity that can occur from the use of selected chemotherapy agents [1,4]. PPE is typically preceded by dysesthesia and progresses to burning pain, diffuse erythema, and ultimately desquamation. Some literature suggests PPE is dose-related and usually occurs within one to twenty one days of high-dose pulse therapies or presents after several months of continuous low-dose therapies [5]. Its incidence depends upon the drug, its dosage, and its manner of administration, specifically the period of time off therapy between doses, and duration of an infusion. HFS should not be confused with hand-foot skin reaction (HFSR), cutaneous toxicity associated with the multikinase inhibitors (MKIs) sorafenib and sunitinib. HFSR presents as localized, painful, hyperkeratotic plaques and blisters predominately on palmoplantar pressure points and areas of friction. Current literature suggests that HFSR typically occurs after the second to fourth weeks of treatment with symptoms usually arising before three months of treatment [5-7].

The most notorious chemotherapy drugs known to cause PPE include: capecitabine-up to 74% of patients, 5-fluorouracil-up to 34% with a continuous infusion and 13% receiving bolus injections, liposomal doxorubicin-up to 48% [4]. Other chemotherapy drugs are high-dose interleukin-2, doxorubicin (continuous infusion), cytarabine, hydroxyurea, idarubicin, cyclophosphamide, methotrexate, tegafur, mitozantrone, paclitaxel, mercaptopurine, floxuridine, vinorelbine, and liposomal daunorubicin. From our review of literature, this is the second known occurrence of PPE secondary to trastuzumab [3]. Previous documentations of PPE in patients receiving trastuzumab have usually been in conjunction with other systemic chemotherapy agents, such as taxanes, liposomal doxorubicin, or capecitabine, however, this was not the case in our patient, as this individual developed PPE when receiving monotherapy trastuzumab regime [3,8,9].

The exact mechanism of PPE is unknown, but it is an area of active investigation with several hypotheses put forward. One of these hypotheses postulates that drug accumulation in eccrine sweat glands results in increased concentrations of harmful products damaging the surrounding skin. In severe cases, PPE has been reported to involve the inguinal and axillary regions along with the palms and soles, all areas with a greater presence of eccrine sweat glands. This hypothesis is further supported by reports of an increased concentration of chemotherapeutic agents in sweat, along with separate reports of microscopic damage to eccrine sweat glands in patients with PPE [5,10]. The difference between HFS and HFSR presentations is currently attributed to distinct underlying pathophysiology. The rich capillary networks found at the thickened dermis allow for an increased concentration of MKI agents, which inhibit vascular endothelial growth factor receptors (VEGFRs) and platelet-derived growth factor receptors (PDGFRs), both involved in angiogenesis. It has been assumed that HFSR is localized, rather than diffuse as in HFS, due to microvascular damage in areas experiencing greater mechanical stress along with an inhibited proangiogenic pathway, dampening vasculature repair mechanisms and escalating cellular damage [5,7].

The most effective management strategy of PPE is the interruption of treatment by dose reduction or lengthening the interval between drug administrations. This usually leads to a decrease and resolve in symptoms within one to two weeks [11]. Several different measures have been suggested to prevent PPE symptoms. Some aim to avoid friction, excessive pressure to the skin, and exposure to extreme temperatures, in addition to the application of emollient creams [12,13]. One randomized clinical trial found that prophylactic use of 10% urea cream three times daily and after hand washing reduced the occurrence of PPE when compared to Maposal, an antioxidant ointment [14]. These results were similar to a randomized, open-label trial that concluded the use of prophylactic 10% urea cream reduced the occurrence of HFSR as well as extended the time to first HFSR onset in patients treated with sorafenib [15]. However, blinded, randomized, placebo-controlled trials to determine the benefit of ureas-based creams in preventing HFS and HFSR are still warranted. Steroids and agents like dimethylsulfoxide are sometimes effective for various grades of PPE, although none have been evaluated by randomized clinical trials [12,13]. Numerous case reports demonstrate the successful use of oral pyridoxine (vitamin B6) at higher doses. One such report stated the effective use of pyridoxine 100 mg thrice daily [16], contrarily, another randomized, double-blinded study showed no benefit with the use of pyridoxine for PPE associated with capecitabine [17]. In a prospective, non-randomized study by Mangili et al. on PPE, patients treated with ice pack application to the extremities showed a statistically significant reduction in the incidence and severity of erythrodysesthesia [18]. Although the efficacy of cold packs needs to be proven prospectively in a controlled and randomized trial, Mangili et al. suggested that the introduction of the regional cooling strategy, together with correct instructions to patients regarding advised behavior, could play a fundamental role in reducing the incidence of this side effect. While not seen in prior reports, we propose that an interaction between radiation and trastuzumab heightened the toxicity of erythrodysesthesia and believe further exploration is warranted.

## Conclusions

PPE is a common cutaneous toxicity in patients receiving treatment with several chemotherapeutic agents as well as targeted therapies like tyrosine kinase inhibitors (TKIs), though is not typically associated with trastuzumab. The incidence of a severe grade PPE is low; however, it can have a significant influence on a patient's quality of life. Prevention and treatment measures, though effective, still need their efficacy assessed through controlled and randomized studies. The authors urge clinicians to expect and watch out for early signs of dermatologic desquamation in lone trastuzumab therapy.
